# Kounis Syndrome Triggered by Hymenoptera Stings and Exacerbated by Streptokinase

**DOI:** 10.1016/j.jaccas.2026.108921

**Published:** 2026-06-18

**Authors:** Siew Yap Chai, Kai Soon Liew, Kok Ming Soh, Seetha Devi Subramanian, Kenneth Kay Leong Khoo, Dharmaraj Karthikesan

**Affiliations:** Cardiology Department, Hospital Sultanah Bahiyah, Alor Setar, Kedah, Malaysia

**Keywords:** allergic angina, hymenoptera sting, Kounis syndrome, mast cell degranulation, ST-segment elevation myocardial infarction (STEMI), streptokinase

## Abstract

**Background:**

Kounis syndrome (KS) is an allergy-mediated acute coronary syndrome that is frequently under-recognized and potentially life threatening.

**Case Summary:**

A 52-year-old man developed an inferior ST-segment elevation myocardial infarction following a bee swarm attack. Standard thrombolysis with streptokinase triggered severe hemodynamic collapse and systemic hemorrhage. Upon recognizing KS, management was shifted to steroids and antihistamines, leading to complete recovery. Recurrence was documented 8 years later following an ant bite.

**Discussion:**

This case illustrates how standard acute coronary syndromes treatments, such as antigenic thrombolytics, can inadvertently exacerbate the allergic cascade in KS. It highlights the need to maintain a high index of suspicion when cardiac symptoms follow an allergic trigger.

**Take-Home Messages:**

Avoid antigenic thrombolytics when KS is suspected to prevent iatrogenic clinical deterioration. Primary percutaneous coronary intervention and early allergy management are the preferred clinical strategies.

## Visual Summary

**Figure:**
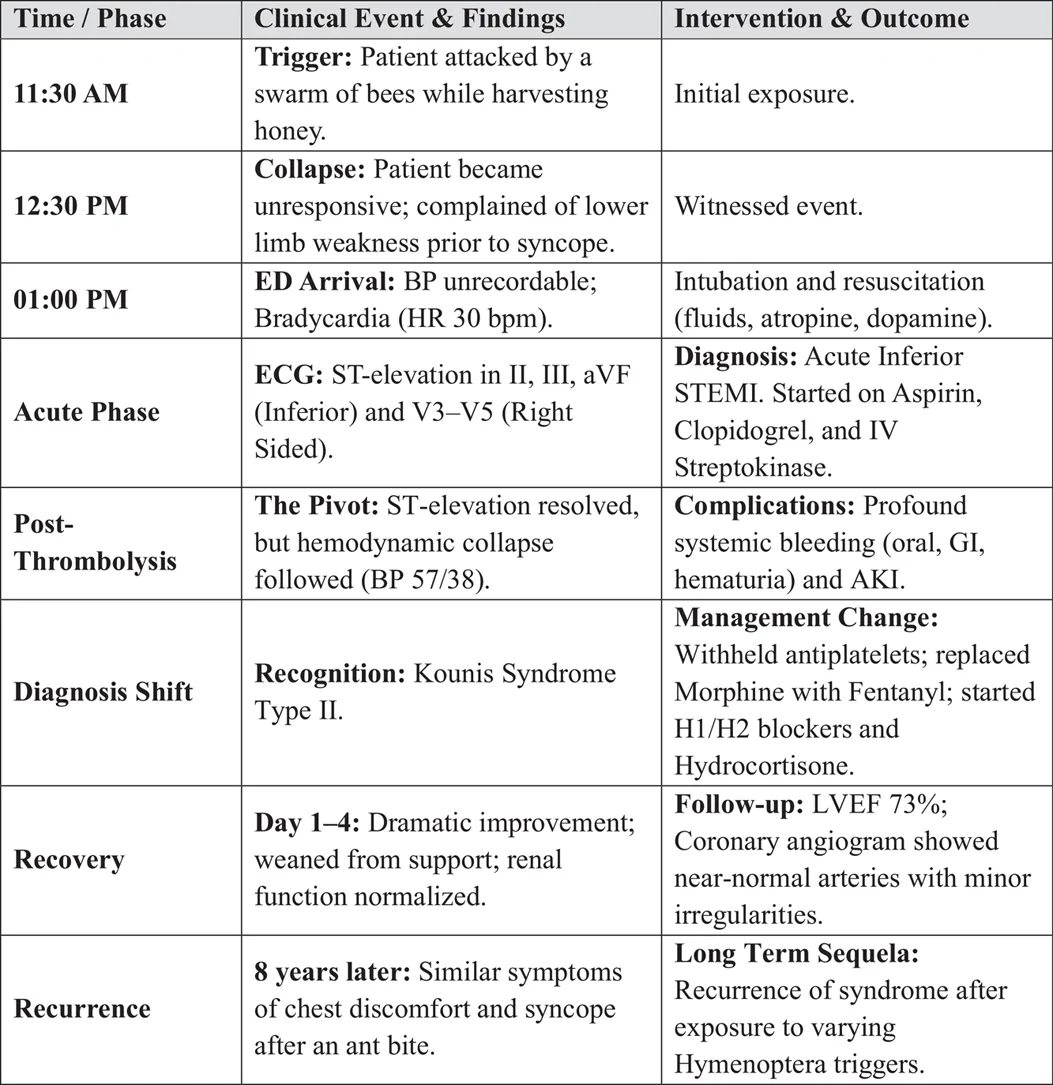

The intersection of allergy and cardiology often creates a diagnostic blind spot in the emergency setting. Kounis syndrome (KS), or allergic angina, remains an under-recognized cause of acute coronary syndromes (ACS). This case highlights the complexity of managing KS when standard ACS protocols, especially the use of streptokinase, inadvertently worsen the underlying allergic cascade.

## Case Presentation

A 52-year-old man, a chronic smoker with no known medical illnesses, presented to our emergency department after an attack by a swarm of bees. He was known to have prior allergic reaction to a hornet sting 1 year earlier, which resulted in periorbital swelling and breathlessness treated with epinephrine, hydrocortisone, and chlorphenamine.

On this occasion, while harvesting wild honey at 11:30 am, he was stung multiple times. One hour later, he collapsed. Witness reports noted no chest pain, shortness of breath, or seizures before the collapse, although the patient had reported lower limb weakness before losing consciousness. Upon arrival to the emergency department at 1:00 pm, he was in profound shock: his blood pressure was unrecordable, and he was bradycardic at 30 beats/min. Physical examination revealed no signs of a systemic allergic response, with an absence of rash, swelling, or visible sting marks.

## Initial Management and Diagnosis

The patient was immediately intubated and resuscitated with intravenous fluids, atropine, and a dopamine infusion. His vitals improved to a heart rate of 120 beats/min and a blood pressure of 101/57 mm Hg. An initial electrocardiogram (ECG) revealed ST-segment elevation in leads II, III, and aVF, with reciprocal changes in leads I and aVL. ST-segment elevation in V_4_R-V_6_R showed ST-segment elevation in V_3_-V_5_, leading to a diagnosis of acute inferior ST-segment elevation myocardial infarction with right ventricular involvement (Figure 1, Figure 2, Figure 3).

**Figure 1:**
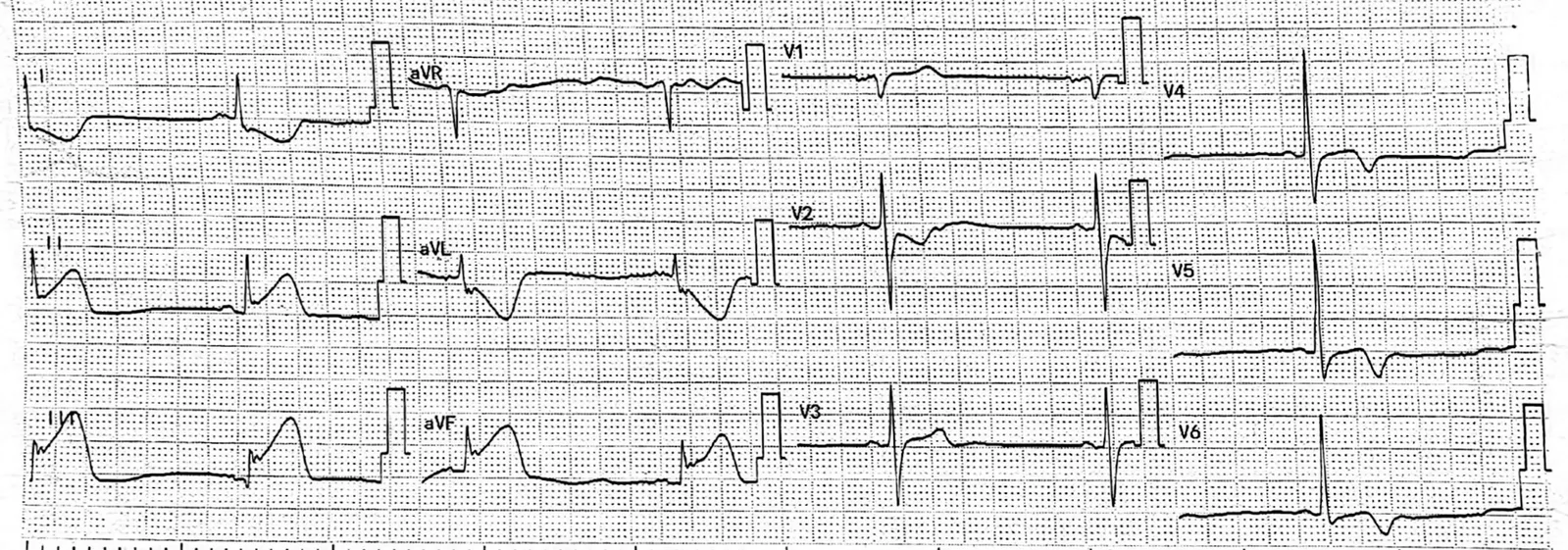
12-Lead Electrocardiogram on Initial Presentation to the Emergency Department in 2014

**Figure 2:**
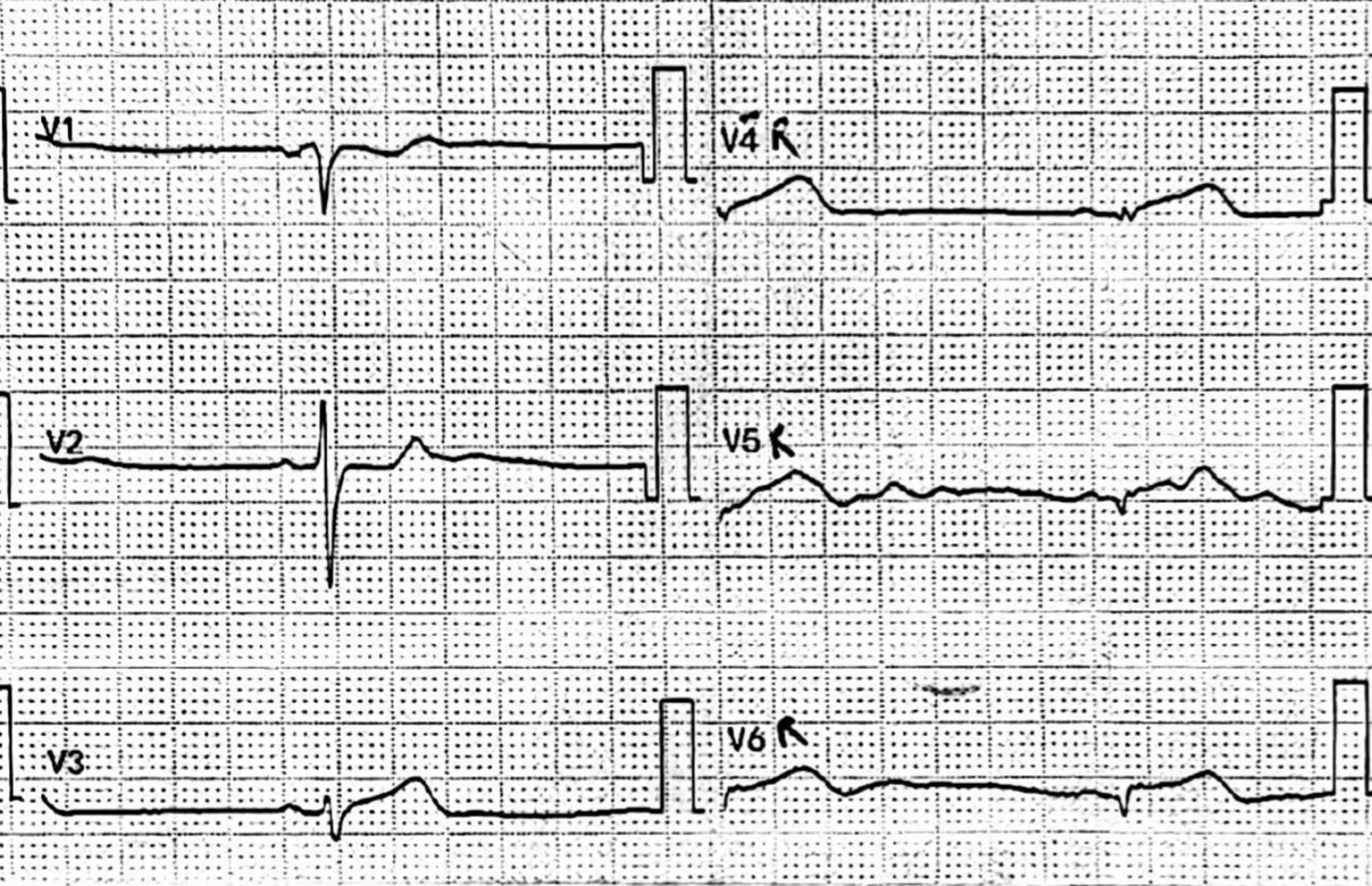
Right-Side Electrocardiogram

**Figure 3:**
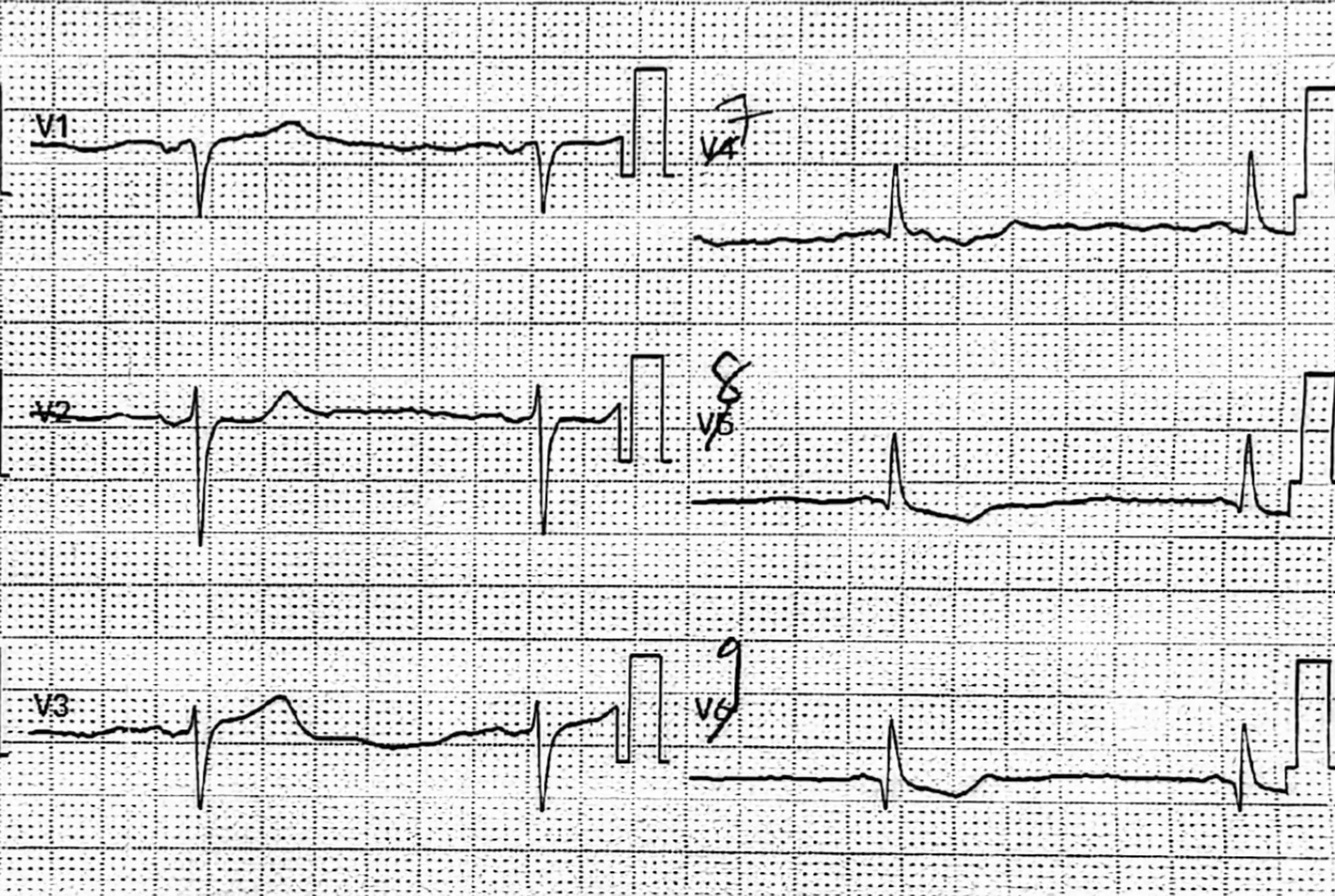
Posterior Electrocardiogram

Following local protocols, he received oral aspirin and clopidogrel, and was started on thrombolysis with intravenous streptokinase; primary percutaneous coronary intervention was unavailable. While the ST-segment elevation resolved 30 minutes post-thrombolysis, the patient's condition deteriorated upon transfer to the cardiac care unit.

## Complications and Revised Diagnosis

Despite maximum triple inotropic support, the patient's blood pressure plummeted to 57/38 mm Hg. He began bleeding from the nasal and oral cavities, accompanied by active upper gastrointestinal bleeding and hematuria. Laboratory results showed a significantly prolonged activated partial thromboplastin time (>180 seconds), while the international normalized ratio remained between 1.2 and 1.4.

At this juncture, the clinical team recognized that the patient was likely experiencing KS, exacerbated by the antigenic nature of streptokinase. We immediately withheld antiplatelets and initiated blood transfusions. Morphine infusion is known to trigger mast cell degranulation, hence we replace it with fentanyl infusion. He was started on a regimen of intravenous H1 and H2 antihistamines and hydrocortisone.

## Outcome and Follow-Up

The clinical response was dramatic. Within 24 hours, the patient was weaned from inotropes and mechanical ventilation. His renal function, which had declined because of hypoperfusion, normalized within 4 days. An echocardiogram on day 3 showed a normal left ventricular ejection fraction of 73% with no regional wall motion abnormalities. A subsequent coronary angiogram revealed near-normal arteries, with only minor irregularities in the mid left anterior descending artery and mid right coronary artery (Figure 4, Figure 5, Figure 6).

**Figure 4:**
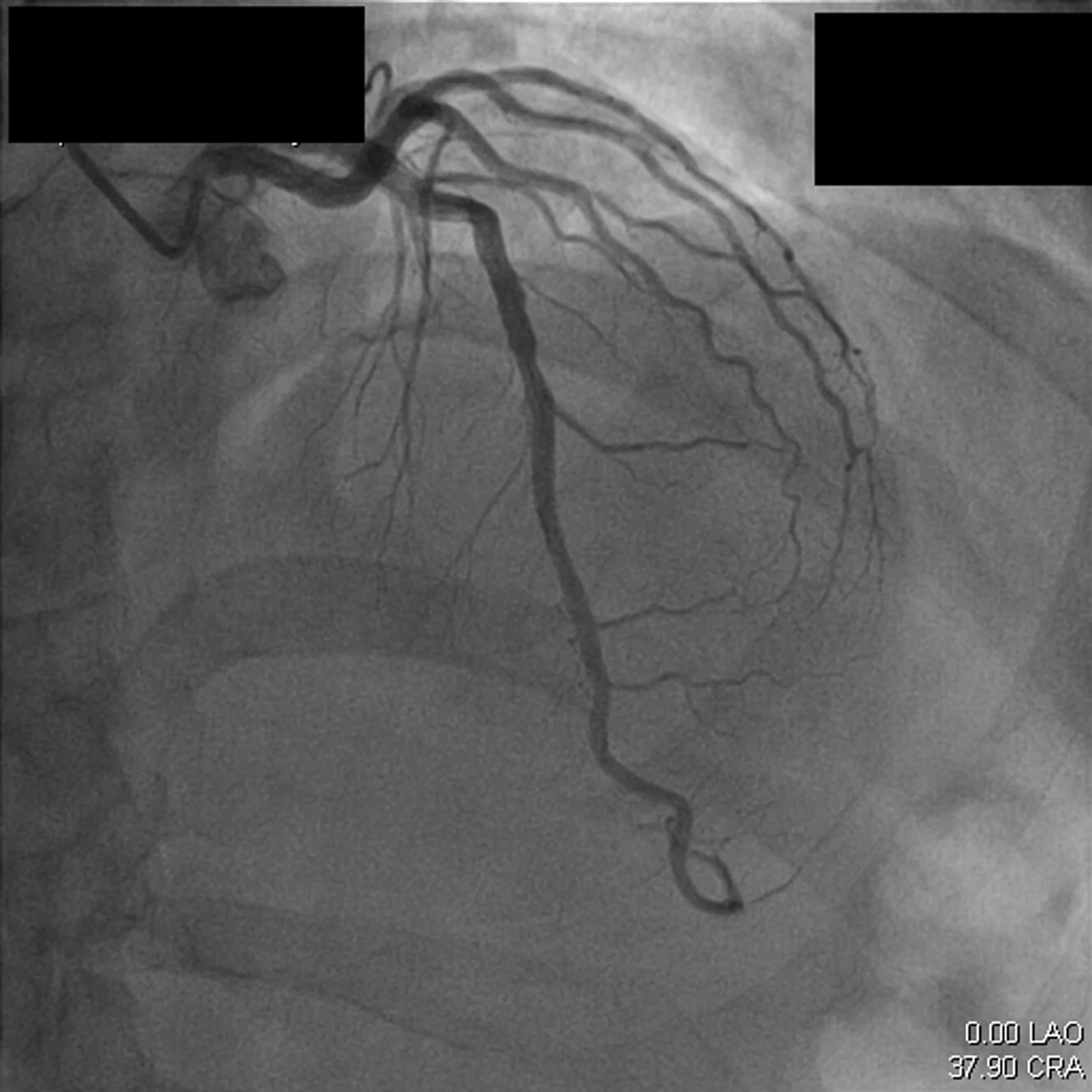
Left Anterior Descending Artery

**Figure 5:**
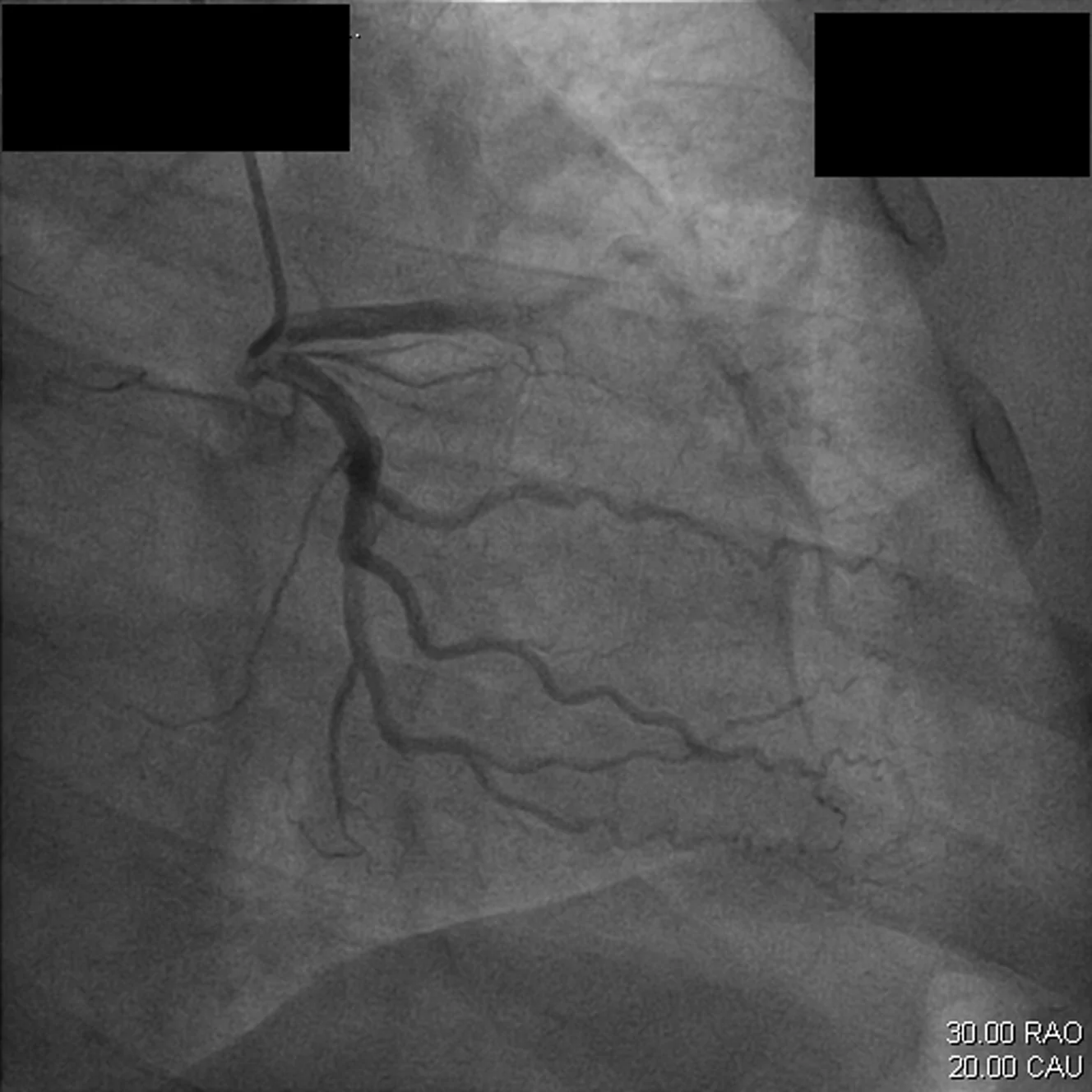
Left Circumflex Artery

**Figure 6:**
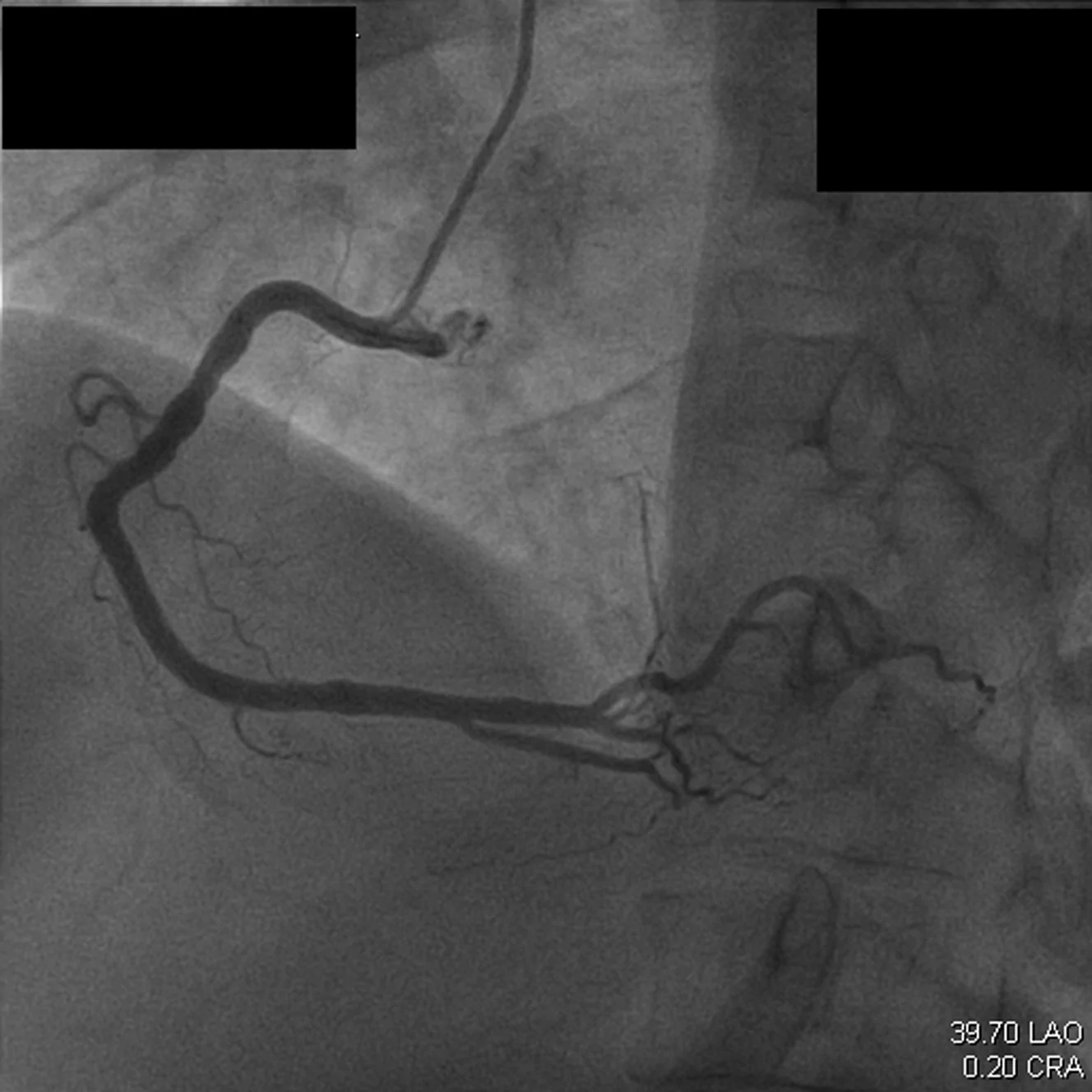
Right Coronary Artery

Eight years later, in July 2022, the patient re-presented with a near-identical clinical syndrome of chest tightness and syncope after being bitten by an arboreal bicolored ant. ECG demonstrated a subtle ST-segment elevation in lead I (Figure 7), accompanied by a sharp rise in troponin levels from 3.8 ng/L to 140.7 ng/L in 1 day and impending coagulopathy. Mindful of his previous severe adverse reaction to standard ACS therapies, the treating team adopted a conservative management strategy, administering only corticosteroids and antihistamines. The patient's symptoms promptly resolved, his troponin levels decreased to 28.1 ng/L on the third day, and he was uneventfully discharged after 3 days of inpatient observation.

**Figure 7:**
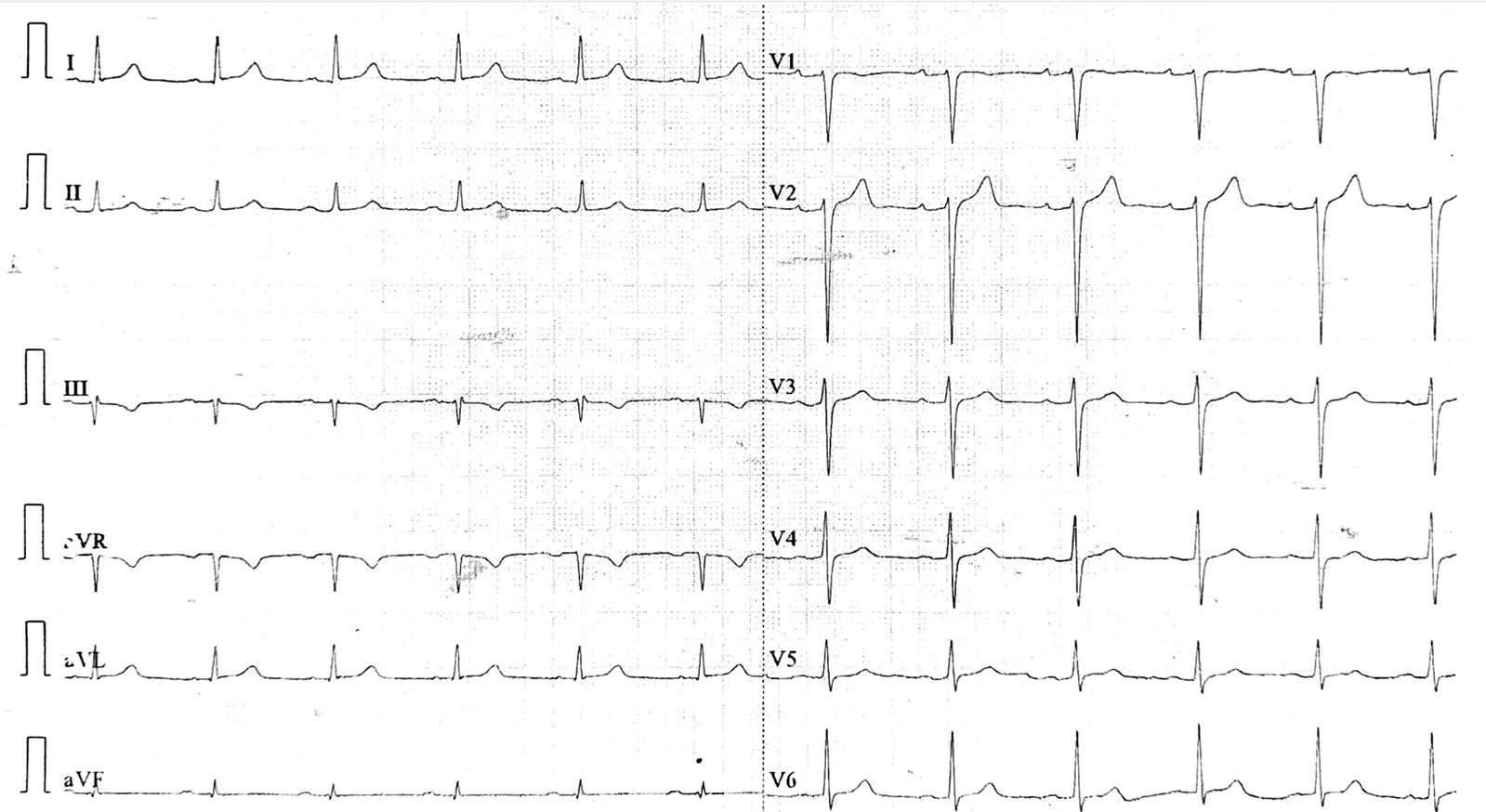
12-Lead Electrocardiogram on Second Presentation to the Emergency Department in 2022

## Discussion

The complex interaction between cardiovascular pathology and immunology are often overlooked in the emergency cardiology domain. Kounis syndrome, also known as allergic angina or allergic myocardial infarction, is a rare but potentially life-threatening condition characterized by the acute onset of chest pain, myocardial infarction, or sudden cardiac death in the setting of allergic or hypersensitivity reactions.^1^ While first formally described by Kounis and Zavras in 1991 more than 3 decades ago,^2^ the recognition and diagnosis of this clinical entity remain challenging and often require a high index of suspicion.

The pathophysiology of KS represents the deadly interaction between immunology and cardiology, where the heart becomes the primary target of a systemic hypersensitivity reaction leading to coronary vasospasm, destabilization of atherosclerotic plaques, or thrombosis within coronary stents. Current classification outlines KS into 3 subtypes: type I KS is indicated by allergic vasospastic angina in patients without coronary artery disease; type II KS is defined by acute myocardial infarction in patients with preexisting atherosclerosis; and type III KS is classified by stent thrombosis from hypersensitivity reactions to stent materials.^3^

The central mechanism involves the rapid degranulation of mast cells in the coronary smooth muscles and at the atherosclerotic plaques, which releases a potent cascade of inflammatory mediators including histamine, leukotrienes, prostaglandins, platelet-activating factor, tryptase, and chymase.^3^ In the present case, the patient's near-normal coronary artery classifies this as a type I variant, where mediators likely induced severe vasospasm.

Establishing a definitive diagnosis of KS requires a high index of clinical suspicion. Although patients often present with symptoms mimicking conventional ACS, some may have atypical presentations reflecting the multisystemic nature of the disease, such as the presentation of our patient with syncope and bilateral lower limb weakness, which is suggestive of a possible involvement of the cerebral and spinal vasculatures. The cornerstone of the diagnostic approach is the temporal relationship between an allergic trigger and the onset of cardiac symptoms or ECG changes, most commonly inferior ST-segment elevation.^4^ While serum tryptase is the gold-standard biomarker for mast cell activation, the strict timeline for testing due to its short half life^5^ and the logistical challenges of outsourcing the test in some centers often limit its utility in the acute setting. In this patient, serum tryptase was not sent because the diagnosis was made beyond the appropriate timeframe for testing.

A critical lesson from this case is the iatrogenic risk posed by standard ACS treatments. The administration of morphine, which can induce further mast cell degranulation, and the highly antigenic thrombolytic streptokinase likely acted as secondary catalysts for the patient's deterioration. Furthermore, Hymenoptera venom is known to cause coagulopathy,^6^^,^^7^ which compounded the effect of the pharmacological thrombolysis, resulting in severe life-threatening bleeding in this patient.

The role of standard ACS secondary prevention varies significantly across KS subtypes. Although aspirin is generally unnecessary in type I variants given the absence of coronary artery disease, it is strongly recommended alongside statin therapy in type II (plaque rupture) and type III (stent thrombosis) variants.

The long-term follow-up of this case provides us a rare longitudinal insight into the recurrence of KS 8 years after the index event, where the patient presented with almost identical symptoms after an ant bite. This observation reaffirms that patients who experienced KS are at a lifelong risk of recurrence when exposed to similar triggers.

## Conclusions

Kounis syndrome demands an individualized approach that deviates from standard ACS protocols. Our patient's recovery demonstrates that when KS is suspected, the use of antihistamines and corticosteroids is paramount, and primary percutaneous coronary intervention is the preferred revascularization strategy to avoid the antigenic risks of thrombolytics. The current lack of specific guidelines for KS emphasizes the importance of clinical awareness and individualizing treatment based on the specific variant and trigger.

## Funding Support and Author Disclosures

The authors have reported that they have no relationships relevant to the contents of this paper to disclose.

**Take-Home Messages**

• In patients presenting with acute coronary syndrome after an allergic trigger, a high index of suspicion for Kounis syndrome is vital to avoid iatrogenic harm from standard medications like morphine and antigenic thrombolytics.
• Primary percutaneous coronary intervention is the preferred revascularization strategy for suspected Kounis syndrome, as it avoids the risk of compounding a systemic hypersensitivity reaction with potentially antigenic thrombolytic agents.

**Visual Summary:** Clinical Timeline From Anaphylactic Shock to Kounis Syndrome

Time/Phase	Clinical Event and Findings	Intervention and Outcome
11:30 am	Trigger: Patient attacked by a swarm of bees while harvesting honey.	Initial exposure.
12:30 pm	Collapse: Patient became unresponsive; complained of lower limb weakness before syncope.	Witnessed event.
01:00 pm	ED arrival: BP unrecordable; bradycardia (HR: 30 beats/min).	Intubation and resuscitation (fluids, atropine, dopamine).
Acute phase	ECG: ST-segment elevation in II, III, aVF (inferior) and V_3_-V_5_ (right-sided).	Diagnosis: Acute inferior STEMI. Started on aspirin, clopidogrel, and IV streptokinase.
Post-thrombolysis	The pivot: ST-segment elevation resolved, but hemodynamic collapse followed (BP: 57/38 mm Hg).	Complications: Profound systemic bleeding (oral, GI, hematuria) and AKI.
Diagnosis shift	Recognition: Kounis syndrome type II.	Management change: Withheld antiplatelets; replaced morphine with fentanyl; started H1/H2 blockers and hydrocortisone.
Recovery	Days 1-4: Dramatic improvement; weaned from support; renal function normalized.	Follow-up: LVEF 73%; coronary angiogram showed near-normal arteries with minor irregularities.
Recurrence	8 years later: Similar symptoms of chest discomfort and syncope after an ant bite.	Long-term sequela: Recurrence of syndrome after exposure to varying Hymenoptera triggers.

AKI = acute kidney infection; BP = blood pressure; ECG = electrocardiogram; ED = emergency department; HR = heart rate; GI = gastrointestinal; IV = intravenous; LVEF = left ventricular ejection fraction; STEMI = ST-segment elevation myocardial infarction.
